# Depression as a moderator of STAIR Narrative Therapy for women with post-traumatic stress disorder related to childhood abuse

**DOI:** 10.1080/20008198.2017.1377028

**Published:** 2017-10-13

**Authors:** Marylene Cloitre, Donn W. Garvert, Brandon J. Weiss

**Affiliations:** ^a^ National Center for PTSD, Veterans Affairs Palo Alto Health Care System, Palo Alto, CA, USA; ^b^ Department of Psychiatry and Behavioral Sciences, Stanford University School of Medicine, Palo Alto, CA, USA

**Keywords:** Depression, PTSD, childhood abuse, skills training, psychotherapy, Depresión, TEPT, maltrato Infantil, entrenamiento en habilidades, psicoterapia, 抑郁, 创伤后应激障碍, 儿童期虐待, 技能训练, 心理治疗, • Severe depression is a moderator of treatment outcome among women with PTSD and childhood abuse.• Those with severe depression obtained greater decreases in PTSD symptoms and more consistency in symptom improvement when provided with coping skills in combination with trauma-focused work (STAIR Narrative Therapy) as compared to treatments that provided only one or the other type of intervention.• Among those with low levels of depression, PTSD outcomes did not differ by treatment.• Skills training that supports behavioral activation such as engaging in physical, social, or pleasurable activities may be particularly beneficial to individuals with PTSD and comorbid depression.• Better understanding is needed of the underlying mechanisms which contribute to the severe, persistent, and relapsing depression associated with childhood abuse.

## Abstract

**Background**: Depression among those who have experienced childhood abuse is associated with earlier onset, more persistent and severe symptoms, more frequent relapse, and poorer treatment outcomes across a variety of psychiatric disorders. In addition, individuals with a history of childhood abuse are more likely to develop post-traumatic stress disorder (PTSD) co-occurring with depression.

**Objective**: This study evaluated whether severity of depression moderated the outcome in a PTSD treatment for childhood abuse survivors. Specifically, we assessed whether individuals with significant depression obtained better outcomes when provided with a two-module treatment which included a skills training component with behavioral activation interventions, Skills Training in Affective and Interpersonal Regulation (STAIR) followed by a trauma-focused component, Narrative Therapy, as compared to two control conditions where one component (STAIR or Narrative Therapy) was replaced with Supportive Counseling.

**Method**: Participants were 104 women with PTSD related to childhood abuse. Participants were randomized into three conditions: (1) STAIR plus Narrative Therapy (SNT), (2) STAIR plus Supportive Counseling (SSC), and (3) Supportive Counseling plus Narrative Therapy (SCNT). Clinician-Administered PTSD Scale for DSM-IV (CAPS-IV) PTSD symptom severity was assessed at pre-treatment, post-treatment, and 3 and 6 month follow-up.

**Results**: Participants with severe depression showed superior PTSD symptom reduction following SNT, while those in the other two conditions experienced a loss of improvement after treatment ended. A similar finding was obtained among those with moderate depression, while among those with low levels of depression, outcomes did not differ across the three treatment conditions.

**Conclusions**: Childhood abuse survivors with severe depression obtained superior outcomes in a treatment that combined skills training with trauma-focused work. Skills packages which contain behavioral activation interventions in combination with trauma-focused work may be particularly beneficial for patients with childhood abuse and severe depression.

## Abstract (es)

**Planteamiento**: La depresión entre los que han sufrido malos tratos en la infancia se asocia con un inicio más temprano, síntomas más persistentes y graves, recaídas más frecuentes y peores resultados de tratamiento en una variedad de trastornos psiquiátricos. Además, las personas con abuso infantil son más propensas a desarrollar un TEPT concurrente con la depresión.

**Objetivo**: Este estudio evaluó si la gravedad de la depresión moderaba el resultado en un tratamiento de TEPT para sobrevivientes de maltrato infantil. Específicamente, evaluamos si los individuos con depresión significativa obtenían mejores resultados cuando se les proporcionaba un tratamiento de dos módulos que incluía un componente de entrenamiento en habilidades con intervenciones de activación conductual, Entrenamiento de habilidades en regulación afectiva e Interpersonal (STAIR, siglas en inglés de *Skills Training in Affective and Interpersonal Regulation*), seguido de un componente centrado en el trauma, Terapia Narrativa (TN), en comparación con dos condiciones de control en las que un componente (el STAIR o la TN) se sustituía por terapia de apoyo (TA).

**Métodos**: Los participantes fueron 104 mujeres con TEPT relacionado con maltrato infantil. Los participantes fueron asignados al azar a tres condiciones: (1) STAIR más Terapia Narrativa (STN) (2) STAIR más terapia de apoyo (STA) y (3) terapia de apoyo más Terapia Narrativa (TATN). Se evaluó la gravedad de los síntomas de TEPT con el CAPS-IV al inicio del tratamiento, después del tratamiento y en el seguimiento a los 3 y 6 meses.

**Resultados**: Los participantes con depresión grave mostraron una mayor reducción de los síntomas de TEPT después de la Terapia Narrativa de STAIR, mientras que los de las otras dos condiciones experimentaron una pérdida de mejoría una vez finalizado el tratamiento. Se encontró un resultado similar entre los que tenían depresión moderada, mientras que entre los que tenían un nivel bajo de depresión, los resultados no difirieron entre las tres condiciones de tratamiento.

**Conclusión**: Los supervivientes de maltrato infantil con depresión grave obtuvieron resultados superiores en un tratamiento que combina entrenamiento de habilidades con el trabajo centrado en el trauma. Los paquetes de habilidades que contienen intervenciones de activación conductual en combinación con el trabajo centrado en el trauma pueden ser particularmente beneficiosos para pacientes con abuso infantil y depresión grave.

## Abstract (zh)

**背景**：在经历过儿童期虐待的人中，抑郁与很多精神性障碍的更早的起病、更加持久、更严重、更频繁的复发、更差的治疗效果有关。并且，有童年期虐待经历的人更可能发展出PTSD并发抑郁。

**目标**：本研究评估了抑郁的严重程度是否会对童年期虐待的幸存者的PTSD治疗效果有调节作用。我们特别对比了三种疗法中的治疗效果。第一种疗法具有两个模块：包括一个技能训练部分与行为激活干预，情感和人际调节技能训练（STAIR）和紧接着的创伤中心叙述疗法（NT），两个对照疗法中分别将STAIR或者NT由支持性咨询（SC）所替代。我们评估了是否第一种疗法对显著抑郁的个体效果更好。

**方法**：104名因为童年期虐待而患创伤后应激障碍的女性参与者，被随机分成三个水平：（1）STAIR加叙事疗法（SNT）/（2）STAIR加支持性咨询（SSC）/（3）支持性咨询加叙事疗法（SCNT）。CAPS-IV PTSD症状严重性在治疗前、治疗后、3个月和6个月追踪期被测量。

**结果**：使用STAIR叙事疗法的严重抑郁者表现出显著的PTSD症状减少，而其他两个对照组在治疗结束后好转的症状又复发了。中度抑郁的群体也表现出类似的结果，而低水平抑郁的个体没有表现出组间差异。

**结论**：有严重抑郁的儿童期虐待幸存者，在接受以创伤中心治疗和技能训练的联合治疗之后表现出显著的效果。行为激活干预和创伤中心治疗结合在一起的技能训练组合对经历童年期虐待并患有严重抑郁的患者可能有特殊益处。

## 1. Introduction

The general literature on the relationship between childhood abuse and depression indicates that depression among those who have experienced childhood abuse is associated with more severe and persistent symptoms, longer duration, more frequent relapse, and poorer treatment outcome across a variety of psychiatric disorders relative to those without such a history. A recent meta-analysis reported that individuals who had experienced childhood abuse or neglect were twice as likely to have recurrent and persistent depressive episodes (Nanni, Uher, & Danese, 2012), and data from outpatient samples indicate that adults with childhood neglect and abuse experience a greater severity of depression, earlier onset, and longer duration of illness (Friedman et al., 2012; Miniati et al., 2010). Specific to post-traumatic stress disorder (PTSD), individuals who have experienced childhood abuse and develop PTSD are at much greater risk of developing co-occurring depression. For example, a nationally representative US sample found that adolescents with a history of childhood physical or sexual abuse were 2.8 and 2.4 times, respectively, more likely to meet criteria for comorbid PTSD and depression (Kilpatrick et al., 2003). Compared to individuals with either disorder alone, those with both disorders have higher rates of suicide attempts (Oquendo et al., 2005), greater impulsivity (Oquendo et al., 2005), and higher rates of being discharged against medical advice (Holtzheimer, Russo, Zatzick, Bundy, & Roy-Byrne, 2005). Altogether, these data suggest that individuals who experienced childhood abuse who suffer from PTSD co-occurring with depression may be at risk for poorer treatment outcome owing to the chronicity, severity, and relapsing nature of their depression.

There is some evidence to suggest that increasing severity of depression is associated with poorer outcome in PTSD treatment. For example, Taylor et al. (2001) found that the level of depression differentiated between responders and partial responders following cognitive behavioral therapy (CBT) for PTSD related to road traffic collisions, with greater depression related to worse outcome. Hagenaars, Van Minnen, and Hoogduin (2010) found that patients with current or past depression exhibited more PTSD symptoms following prolonged exposure therapy. Depressive symptoms have also been found to be predictive of treatment dropout from CBT for PTSD (Zayfert et al., 2005). Markowtiz et al. (2015) found that the rate of dropout for prolonged exposure therapy was nine times higher among patients with comorbid PTSD and major depressive disorder than in non-depressed patients. Notably, the impact of depression on outcome in PTSD treatment is similar in other disorders, where co-occurring depression has been shown to negatively impact the course of treatment and outcome for social anxiety disorder (Chambless, Tran, & Glass, 1997; Erwin, Heimberg, Juster, & Mindlin, 2002), obsessive–compulsive disorder (Abramowitz, 2004), and eating disorders (Berkman, Lohr, & Bulik, 2007; Löwe et al., 2001).

Given the substantial comorbidity between PTSD and depression, as well as the impact of depressive symptoms on treatment outcome, investigating the potential benefit of including depression-relevant psychosocial interventions to improve outcome in PTSD treatments is warranted.

We wished to assess whether childhood abuse survivors with PTSD who experienced high levels of depression might have better PTSD outcome when provided with Skills Training in Affective and Interpersonal Regulation (STAIR). This is a coping skills package developed to address emotion regulation difficulties reported by trauma survivors, including both anxiety and depression, and to address interpersonal difficulties, particularly avoidance and feelings of being disconnected from others (Cloitre, Cohen, & Koenan, 2006). STAIR emotion regulation interventions explicitly include ‘down-regulation’ interventions for high affect states (e.g. anxiety, anger) as well as ‘up-regulation’ interventions for low affect states (e.g. depression, numbing). The interventions for the latter include pleasurable activities and exercise scheduling, similar to those recommended in behavioral activation therapy for depression. The interpersonal work includes skills training to improve communication as well as activities to increase social engagement (e.g. hiking, volunteer work), also consistent with behavioral activation recommendations. For these reasons, we hypothesized that STAIR might be a particularly beneficial coping skills program for PTSD patients with depression.

Analyses of data from a randomized controlled trial of CBT for PTSD related to childhood abuse (Cloitre et al., 2010) were performed to examine the relationship between type of treatment and severity of depression in three treatment conditions. The treatment condition of interest was one in which STAIR was added to Narrative Therapy, a modified form of prolonged exposure [STAIR plus Narrative Therapy (SNT)]. The treatment was compared to two control conditions, each of which replaced one of the treatment components with Supportive Counseling: STAIR plus Supportive Counseling (SSC) and Supportive Counseling plus Narrative Therapy (SCNT). We expected that for those with significant depression, SNT would provide superior PTSD outcomes compared to SCNT, which eliminated the skills training but controlled for treatment duration, number of sessions, and therapist contact with Supportive Counseling. We also expected SNT to be superior to the SSC condition, which might provide some benefits owing to skills training but would be weaker than SNT given the absence of trauma-focused work, a type of intervention well known for its effectiveness in reducing PTSD symptoms.

## 2. Methods

### 2.1. Procedures

The randomized controlled study included a total of 104 women, between the ages of 18 and 65 years, who had a primary diagnosis of Diagnostic and Statistical Manual of Mental Disorders, 4th Edition (DSM-IV)-defined PTSD related to childhood sexual abuse and/or physical abuse by a caretaker or person in authority over them before the age of 18 years. The exclusion criteria used in this study were substance dependence not in remission for at least 3 months, psychotic symptoms at the time of assessment, significant cognitive impairment, untreated bipolar disorder, and acute suicidality in the previous 3 months that required hospitalization or referral to the emergency room. If participants were receiving psychotherapy or psychopharmacological treatment they were allowed to continue this treatment as long as the treatment had been ongoing for at least 3 months and the psychotherapy was not PTSD focused. Study participants were randomly assigned to one of three conditions: STAIR plus Narrative Therapy (SNT), STAIR plus Supportive Counseling (SSC), and Supportive Counseling plus Narrative Therapy (SCNT).

The study was approved by the local ethics committee, written informed consent was obtained, and the study was registered at ClinicalTrials.gov (identifier: NCT01488539).

### 2.2. Treatment conditions

SNT is a two-module, 16-session protocol treatment provided weekly, where module 1, comprised of eight sessions, focuses on skills training (STAIR), and module 2, the final eight sessions, introduces the Narrative Therapy. The skills training module provided four sessions on emotion regulation (emotional awareness, emotion regulation, distress tolerance, and acceptance of feelings) followed by social skills training including social engagement, effective assertiveness, and interpersonal flexibility regarding expectations and behaviors. The Narrative Therapy component followed the prolonged exposure protocol, with two modifications: (1) meaning analysis was introduced after the exposure, and (2) *in vivo* exposure to abuse-related-specific stimuli was replaced by skills practice in challenging interpersonal and social situations. Imaginal exposure included narratives of both childhood and adulthood traumas, reviewed in order of distress rating and importance to the participant. The Supportive Counseling component in the other two treatment conditions was comprised of client-directed Supportive Counseling directed at life problems related to abuse history. Skills training and narratives of trauma memories were excluded. (See Cloitre et al., 2010 for more details.)

### 2.3. Measures

#### 2.3.1. Clinician-Administered PTSD Scale for DSM-IV (CAPS-IV)

The CAPS-IV is a clinician-administered semi-structured clinical interview that evaluates the frequency and intensity of each of the 17 symptoms of PTSD on a five-point scale, with scores ranging from 0 = ‘not at all’ to 4 = ‘extremely’ (Blake et al., 1995). The CAPS-IV was used to determine the presence of a PTSD diagnosis, a requirement for enrollment into the study. Diagnosis was made following the DSM-IV 1/2 rule, where a symptom was counted as present if the frequency rating was 1 or more and the severity rating was 2 or more. The CAPS-IV was administered by MA-level clinicians trained to reliability (kappa = .80 for PTSD diagnosis) by a gold-standard rater (MC).

#### 2.3.2. Structured Clinical Interview for DSM-IV (SCID)

The SCID is a semi-structured interview for determining the major DSM-IV Axis I diagnoses (First, Spitzer, Gibbon, & Williams, 2002). The SCID was used to establish the presence of major depressive episodes, current dysthymia, and other major anxiety and mood disorders. The SCID was administered by MA-level clinicians trained to reliability (80% agreement on all diagnoses per patient) with a gold-standard rater (MC).

#### 2.3.3. PTSD Symptom Scale Self-Report (PSS-SR)

The PSS-SR is a brief self-report instrument which assesses the severity of each of the 17 PTSD symptoms outlined in the DSM-IV (Foa, Riggs, Dancu, & Rothbaum, 1993). PTSD symptom severity was measured on a five-point Likert scale ranging from 0 = ‘not at all’ to 4 = ‘extremely’. The PSS-SR was used in the current study to assess change over five points in time: baseline, mid-treatment (after session 8), post-treatment (after session 16), 3 month follow-up, and 6 month follow-up.

#### 2.3.4. Beck Depression Inventory (BDI)

The BDI-II is a brief self-report instrument which assesses depression severity (Beck, Steer, & Brown, 1996). Depression severity was assessed at five points in time during the study: baseline, mid-treatment, post-treatment, 3 month follow-up, and 6 month follow-up. For the purposes of this paper, baseline depression severity scores were categorized in the following way: 0–13, minimal; 14–28, moderate; and 29–63, severe (Beck et al., 1996).

### 2.4. Statistical analyses

The primary analysis was a mixed-effects model for longitudinal data using an intention-to-treat sample (using all available data from all randomized participants) and restricted maximum likelihood estimation.. All missing data were missing at random. All outcomes after the baseline assessment were modeled as functions of time, treatment condition, depression severity categorization at the baseline assessment, and the interaction of these variables. Time was coded in terms of linear, quadratic, and cubic growth to examine non-linear trends. Furthermore, time was treated as a continuous variable, and each observation was coded relative to the baseline and post-treatment assessments, which were coded as 0 and 1, respectively. The model controlled for age (mean-centered). Finally, the model allowed for random variation for each participant’s intercept, linear growth (slope), quadratic growth (acceleration/deceleration), and cubic growth (rate of change in acceleration/deceleration). The model used an unstructured covariance structure for the within-individual error covariance structure. The unstructured covariance structure does not require any assumption in the error structure. A model using variance components for the assumed covariance structure was evaluated, but this model did not yield a significantly better fit to the data when examining the −2 log likelihood, Akaike’s information criterion, or the Bayesian information criterion. Adequate model fit was assessed by visual inspection of the distribution of the standardized error terms and the random effects (all are assumed to have a normal distribution). The model was fitted using the SAS PROC MIXED® software, version 9.3 (SAS Institute, Cary, NC, USA). The longitudinal analyses were supplemented by cross-sectional comparison of means and differences in treatment effect slopes in the follow-up period. Effect sizes using Cohen’s *d* (Cohen, 1992) were calculated to assess the clinical significance of change from pre-treatment to 6 month follow-up, as well as across the three treatment conditions.

## 3. Results

### 3.1. Participant characteristics

Participants had a mean age of 36.38 (*SD* = 9.39) years. Most of the sample identified as Caucasian (35.6%, *n* = 37), followed by African-American (27.9%, *n* = 29), Hispanic (26.0%, *n* = 27), and other (10.6%, *n* = 11). The majority of the sample reported that they had graduated from college or attended college beyond an undergraduate degree (52.9%, *n* = 55), 34.6% (*n* = 36) had attended college but did not graduate, 7.7% (*n* = 8) had completed high school but did not attend college, and 4.8% (*n* = 5) did not complete high school. The majority of participants reported some employment (56.7%, *n* = 59), with 30.8% (*n* = 32) being employed full-time (≥35 hours per week) and 26.0% (*n* = 27) indicating that they were employed part-time (<35 hours per week). Sociodemographic characteristics were similar across the three treatment conditions and details can be reviewed in the original report (Cloitre et al., 2010).

BDI depression severity levels at the baseline assessment were as follows: 24.0% minimal (*n* = 25), 51.0% moderate (*n* = 53), and 23.1% severe (*n* = 24). As determined by the SCID, a majority (73.1%) of the sample carried some type of DSM-IV depressive disorder: over one-third met criteria for current major depressive episode (36.5%, *n* = 38), approximately one-fifth met criteria for dysthymia (22.1%, *n* = 23), and 8.7% (*n* = 9) met criteria for both current major depressive episode and dysthymia. There were no differences in characteristics of depressive disorders across the three treatment conditions (Table 1).

**Table 1.** Depression characteristics by treatment condition at baseline.

	SNT (*n* = 33)	SSC (*n* = 38)	SCNT (*n* = 33)
Current MDE diagnosis	14 (42.4)	12 (31.6)	12 (36.4)
Current dysthymia diagnosis	7 (21.2)	11 (28.9)	5 (15.2)
Current dysthmia and MDE	3 (9.1)	4 (10.5)	2 (6.1)
BDI-II total score	18.53 ± 10.03	21.11 ± 8.80	22.13 ± 10.59
BDI-II level of severity			
Minimal	11 (34.4)	6 (15.8)	8 (25.0)
Moderate	14 (43.8)	25 (65.8)	14 (43.8)
Severe	7 (21.9)	7 (18.4)	10 (31.3)

Data are shown as *n* (%) or mean ± *SD*.

SNT, STAIR plus Narrative Therapy; SSC, STAIR plus Supportive Counseling; SCNT, Supportive Counseling plus Narrative Therapy; MDE, major depressive episode; BDI, Beck Depression Inventory.

A comparison of dropout rate by level of depression indicated that the relationship between moderate depression and dropout approached significance (*p* = .053). Among those with minimal depression, 16% (*n *= 4) dropped out of treatment, and the percentage of dropouts distributed across the three conditions was as follows: SNT = 0%, SSC = 4%, and SCNT = 12% (the percentages within the treatment conditions were 0%, 2.6%, and 9%, respectively). Among those with moderate depression, 37.7% (*n *= 20) dropped out, with the percentage of dropouts distributed across the three conditions being SNT = 7.5%, SSC = 17%, and SCNT = 13.2% (within the treatment conditions: 12%, 18.4%, and 27.3%, respectively). Among those with severe depression, 16.7% (*n* = 4) dropped out, with the percentage distributed across conditions being SNT = 4.2%, SSC = 0%, and SCNT = 12.5% (within the treatment conditions: 3%, 0%, and 9%, respectively).

### 3.2. Main effect of depression severity

There was a main effect of depression severity as measured by the BDI-II at the baseline assessment (*F*[2, 96] = 23.91, *p* < .001), where those who were severely or moderately depressed had significantly higher PTSD severity (PSS-SR) scores than those who were minimally depressed at the baseline assessment (*M* = 19.30, *p* < .001, and *M* = 7.64, *p* = .002, respectively). Furthermore, those who were severely depressed had significantly higher PTSD severity scores than those who were moderately depressed (*M* = 11.65, *p* < .001). PTSD severity at baseline did not differ significantly by treatment condition (*F*[2, 96] = 0.30, *p* = .742).

### 3.3. Interaction effect of depression severity and treatment condition on PTSD severity over time

**Figure 1.**
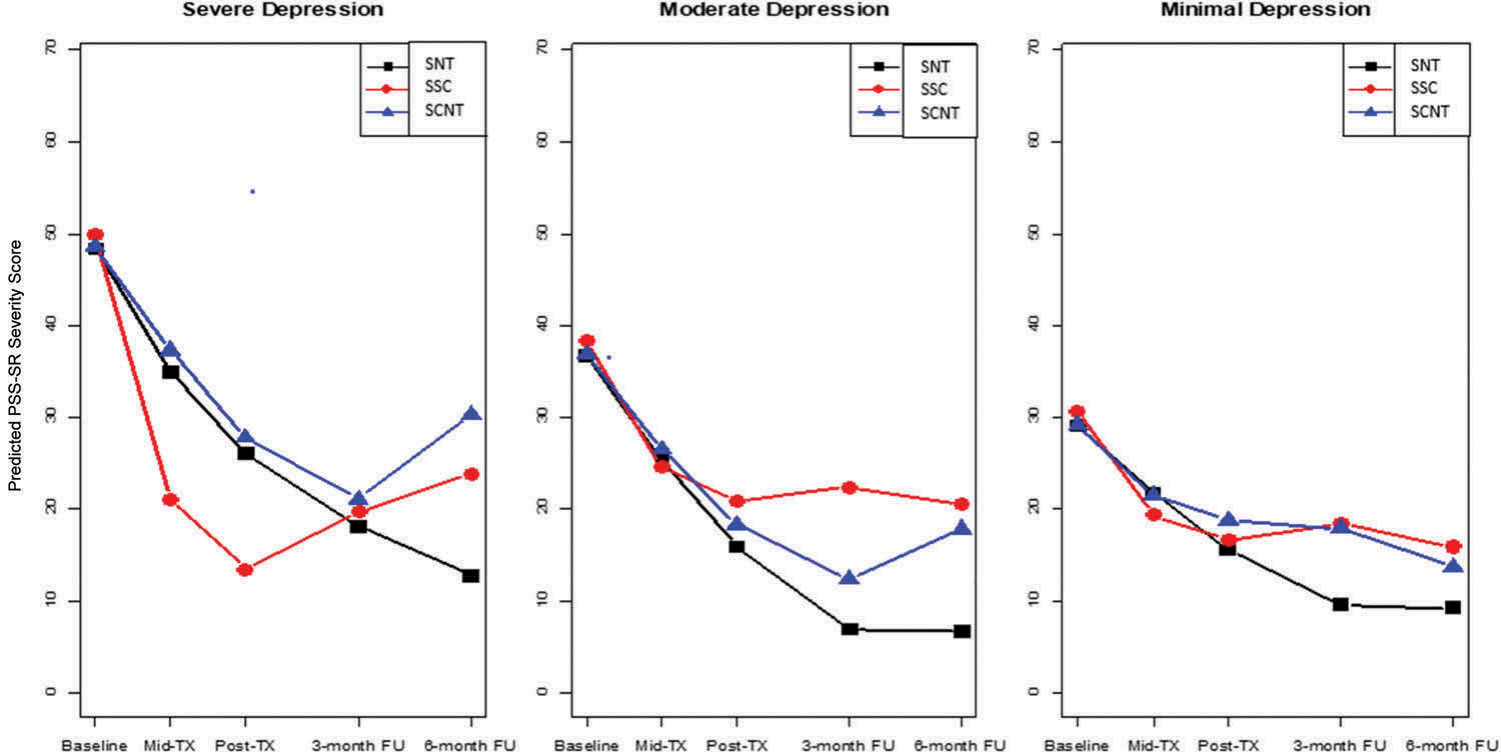
Predicted DSM-IV post-traumatic stress disorder (PTSD) severity [PTSD Symptom Scale Self-Report (PSS-SR)] scores over time by Beck Depression Inventory-II (BDI-II) depression severity and treatment condition. SNT, STAIR plus Narrative Therapy; SSC, STAIR plus Supportive Counseling; SCNT, Supportive Counseling plus Narrative Therapy; TX, treatment; FU, follow-up.

Three different types of interaction effects were evaluated: treatment condition, depression severity, and one of the three respective types of growth over time (linear, quadratic, and cubic growth). Table 2 contains the predicted mean of PTSD severity (PSS-SR) scores at the major assessment points. The predicted trajectories of treatment condition within depression severity levels can be found in Figure 1. The interaction of treatment condition, depression severity, and linear growth was significant (*F*[8, 801] = 2.86, *p* = .004). There was also a significant interaction of treatment condition, depression severity, and quadratic growth (*F*[8, 801] = 2.88, *p* = .004). Finally, there was a significant interaction of treatment condition, depression severity, and cubic growth (*F*[8, 801] = 2.51, *p* = .011). These findings indicate that for each combination of treatment condition and depression severity, PTSD severity changed differently over time for each growth term evaluated.

**Table 2.** Predicted DSM-IV post-traumatic stress disorder (PTSD) severity means and standard errors as measured by the PTSD Symptom Scale Self-Report (PSS-SR) at major assessment points for each treatment condition by depression severity at baseline.

	SNT (*n* = 33)			SSC (*n* = 38)			SCNT (*n* = 33)			Between-group *d* at 6 month FU		
	*M*	*SE*	Within-group *d* pre-6 month FU	*M*	*SE*	Within-group *d* pre-6 month FU	*M*	*SE*	Within-group *d* pre-6 month FU	SNT vs SSC	SNT vs SCNT	SSC vs SCNT
**Severe depression**												
Baseline	48.37	2.50		50.02	2.48		48.51	2.38				
Mid-treatment	34.89	4.39		21.02	4.18		37.30	3.76				
Post-treatment	26.10	4.95		13.50	4.67		27.80	4.18				
3 month FU	18.11	5.40		19.64	5.19		21.12	4.61				
6 month FU	12.77	6.89		23.79	6.65		30.25	5.72				
			2.46			1.82			1.26	0.29	0.46	0.17
**Moderate depression**												
Baseline	36.71	2.11		38.37	1.73		36.86	2.07				
Mid-treatment	25.38	3.19		24.67	2.54		26.52	3.22				
Post-treatment	15.92	3.58		20.90	2.87		18.34	3.73				
3 month FU	6.85	4.10		22.42	3.19		12.35	4.50				
6 month FU	6.62	5.75		20.59	4.09		17.86	5.44				
			2.60			1.54			1.64	0.47	0.38	0.09
**Minimal depression**												
Baseline	29.07	2.31		30.73	2.50		29.22	2.45				
Mid-treatment	21.78	3.43		19.31	4.57		21.52	4.17				
Post-treatment	15.60	3.79		16.64	5.26		18.84	4.76				
3 month FU	9.55	4.02		18.42	5.97		17.89	5.87				
6 month FU	9.22	5.06		15.89	7.50		13.70	7.55				
			1.39			1.04			1.09	0.17	0.11	0.05

SNT, STAIR plus Narrative Therapy; SSC, STAIR plus Supportive Counseling; SCNT, Supportive Counseling plus Narrative Therapy; *d*, Cohen’s *d*, where within-group effect sizes were calculated as the standardized difference of the predicted PTSD severity means at baseline and the 6 month follow-up (FU), and the standardization was based on the observed pooled standard deviation of the three treatment conditions at baseline. The pooled standard deviation of PTSD severity at baseline was 14.28.

### 3.4. Difference in treatment effect slopes from post-treatment to 3 and 6 month follow-up

To interpret the interaction effects, we assessed differences in slopes across the three treatment conditions for each level of depression. All three treatment conditions showed significant reductions from pre- to post-treatment at each level of depression with little difference across conditions, with one exception. Among those with severe depression, SSC showed a steeper slope compared to both the SCNT treatment condition (*M* = −15.81, *p* = .009) and the SNT treatment condition (*M* = −14.26, *p* = .032), indicating a more rapid reduction in PTSD severity during that period. The more pronounced differences across conditions occurred during the follow-up phase, as described below (see Figure 1).

#### 3.4.1. Participants with severe depression

See panel 1 of Figure 1. Among those with severe depression, the slopes from post-treatment to the 3 month follow-up for both SNT and SCNT were negative, reflecting a reduction in PTSD severity, while SSC had a positive slope, indicating an increase in PTSD severity. Thus, while the SSC group (no narrative therapy) had shown greater gains during the treatment phase relative to the other two conditions, these were lost during follow-up. The difference in slope (which includes rate as well as direction of change) between SNT and SSC was significant (*M* = −14.13, *p* = .001), as was that between SCNT and SSC (*M* = −12.81, *p* = .001). At 3 month to 6 month follow-up, SNT was the only treatment that had a negative slope, indicating continued reduction in PTSD severity. The positive slopes for SSC and SCNT indicated an increase in symptoms, indicating further loss of improvement in the SSC condition (no narrative work) and loss of improvement for SCNT, the treatment with narrative work but no skills training during the 3–6 month follow-up period. Comparisons of slopes indicated that SNT differed significantly from SCNT (*M* = −14.47, *p* = .017) but the difference between SNT and SSC did not reach significance (*M* = −9.50, *p* = .144). When comparing overall treatment effect slopes from post-treatment to the 6 month follow-up, the SNT treatment condition had significantly greater negative slopes relative to both the SSC and SCNT treatment conditions (*M* = −23.62, *p* < .001, and *M* = −15.78, *p* = .008, respectively).

A comparison of mean PTSD scores at 6 month follow-up across the three conditions indicated that PTSD scores for SNT were lower than for SCNT (*p* = .048), while the scores for SSC (*M* = 23.79) fell in the middle and did not significantly differ from the other two treatment conditions (Table 2). Between-group effect sizes indicated that SNT was clinically superior to both SSC (*d* = .29) and SCNT (*d* = .46).

#### 3.4.2. Participants with moderate depression

See panel 2 of Figure 1. At post-treatment to 3 month follow-up, both SNT and SCNT had significantly greater negative slopes of PTSD severity compared to SSC (*M *= −10.58, *p* < .001, and *M* = −7.58, *p* = .007, respectively). No significant differences were found in treatment effect slopes from the 3 month follow-up to the 6 month follow-up, or from post-treatment to 6 month follow-up.

A comparison of mean PTSD scores at 6 month follow-up across the three conditions for those with moderate depression indicated that SNT scores for PTSD were significantly lower than for SSC (*p* = .047) but did not reach significance when compared to SCNT (*p* = .154) (Table 2). Effect sizes comparing SNT to the other two treatments at 6 month follow-up indicated greater clinical benefit relative to both SSC (*d* = .47) and SCNT (*d* = .47).

#### 3.4.3. Participants with low depression

See panel 3 of Figure 1. No significant differences were found in slopes across the treatment conditions at any time interval. At the 6 month follow-up, there were no significant differences in PTSD severity across conditions.

## 4. Discussion

The general literature has indicated that individuals with a history of childhood abuse are at risk for more chronic and severe depression and poorer treatment outcome. In PTSD studies, childhood abuse survivors are more likely to suffer from depressive disorders, suggesting that they may be at greater risk for poorer outcome compared to individuals without childhood abuse. This study evaluated depression among childhood abuse survivors as a potential moderator of treatment outcome. Specifically, we assessed whether those with severe depression might benefit from a combination treatment of skills training plus trauma-focused work relative to treatment conditions that excluded one or the other treatment component. The results indicated that among those with severe depression, SNT was associated with greater improvement in PTSD severity compared to the other two treatment conditions. Specifically, severely depressed patients who received SNT experienced continuing reduction in PTSD symptoms after the end of treatment and during the follow-up period, while those in the other two treatment conditions experienced significant loss of improvement over time. Overall, SNT was the only treatment condition in which there was never a period of symptom increase at any level of depression (see Figure 1).

Childhood abuse survivors have been characterized as being at risk for not only for severe but also for relapsing depression (e.g. Nanni et al., 2012). It may be that the inclusion of skills along with trauma-focused work in SNT provides some protection against a tendency for relapsing into symptoms of either depression or PTSD after treatment ends, and perhaps particularly when a stressor occurs. STAIR provides emotion regulation skills that focus on the reduction and management of emotional distress which can support resolution of symptoms in times of stress. In addition, and perhaps more relevant to depression, STAIR includes the use of ‘behavioral activation’ strategies in the form of interventions that focus on increasing physical activities, pleasurable activities, and social engagement, all of which are recommended for the treatment of depression.

The results indicated a graded benefit in the use of combined skills plus trauma work. After treatment ended and throughout the follow-up phase, those with severe depression who had received SNT showed a greater rate of PTSD symptom reduction as well as a more consistent course of improvement compared to those in either of the other two treatment conditions. However, even among those with moderate depression, by the 6 month follow-up, PTSD scores for those who had received SNT were statistically lower than those for the skills treatment without exposure (SSC) and lower to a clinically meaningful degree than both treatment conditions (*d* = .47 compared to SSC and *d* = .38 compared to SCNT).

Differences in treatment dropout across the three conditions approached significance (*p* = .053). However, it is notable that dropout was low and equally so among those with low and high depression, while those with moderate depression exhibited the highest dropout rate. It may be that factors related to motivation for treatment among those who have either high or low symptom burden change little once treatment begins, albeit for different reasons, while among those with moderate depression, the burden of treatment and treatment attendance relative to expected benefit may be more variable. In future studies, it would be of interest to conduct assessments that explore curvilinear and other types of relationships between severity of depression and dropout.

A number of limitations should be considered when interpreting the results of this study. First, the sample size was relatively small. Available data at various time-points decreased over time, potentially leading to inflated error terms for predictions over time. Replication with larger samples would be valuable. In addition, the sample was comprised of females who sought treatment for PTSD related to childhood abuse. Given that depression is more prevalent in women and research findings indicate a variety of gender-related effects of depression, it could be that the findings will not generalize. The relative effectiveness of this particular combination treatment among individuals of other genders and with other types of trauma exposure should be explored in future studies.

The reasons why childhood abuse survivors with depression consistently have poorer treatment outcomes in treatments not only for PTSD but for a range of other disorders are unknown. Investigations which systematically explore possible mechanisms of action for these effects are needed. Nevertheless, the findings from this study indicate that level of depression severity is an important consideration in treatment selection and that the negative impact of depression can be alleviated through added interventions such as STAIR. Individuals with minimal or even moderate depression may do well with traditional trauma-focused interventions for PTSD. However, among those with experience of childhood abuse and severe levels of depression, greater benefits may be more likely to be achieved using a combined treatment approach in which skills training is added to trauma-focused work.
